# Oxidative Stress, High Density Lipoproteins and Hidradenitis Suppurativa: A Prospective Study

**DOI:** 10.3390/antiox14081014

**Published:** 2025-08-19

**Authors:** Elisa Molinelli, Camilla Morresi, Maria Luisa Dragonetti, Edoardo De Simoni, Matteo Candelora, Samuele Marasca, Daisy Gambini, Sara Belleggia, Pietro Dragonetti, Giovanni Di Benedetto, Gianna Ferretti, Tiziana Bacchetti, Oriana Simonetti

**Affiliations:** 1 Dermatological Unit, Department of Clinical and Molecular Sciences, Polytechnic University of Marche, 60126 Ancona, Italy; e.molinelli@univpm.it (E.M.); e.desimoni@pm.univpm.it (E.D.S.); matteo.candelora@ospedaliriuniti.marche.it (M.C.); s.marasca@pm.univpm.it (S.M.); daisy.gambini@ospedaliriuniti.marche.it (D.G.); s.belleggia@pm.univpm.it (S.B.); o.simonetti@staff.univpm.it (O.S.); 2 Department of Life and Environmental Sciences, Polytechnic University of Marche, 60126 Ancona, Italy; c.morresi@univpm.it (C.M.); t.bacchetti@univpm.it (T.B.); 3 Department of Experimental and Clinical Medicine, Clinic of Plastic and Reconstructive Surgery, Polytechnic University of Marche, 60126 Ancona, Italy; p.dragonetti@pm.univpm.it (P.D.); giovanni.dibenedetto@ospedaliriuniti.marche.it (G.D.B.); 4 Department of Odontostomatologic and Specialized Clinical Sciences, Research Center of Health Education and Health Promotion, Research Center on Obesity, Polytechnic University of Marche, 60126 Ancona, Italy

**Keywords:** hidradenitis suppurativa, oxidative stress, high-density lipoprotein, paraoxonase-1

## Abstract

Hidradenitis suppurativa (HS) is a chronic, inflammatory, immune-mediated skin disease associated with several comorbidities and vascular risk factors. Oxidative stress, inflammation, and altered high-density lipoprotein (HDL) functions play key roles in inflammatory skin diseases. However, the relationship between these factors and HS is not fully understood. The aim of this study was to investigate the relationship between HS and oxidative stress, inflammation, and HDL functions, focusing on inflammatory markers and HDL-related antioxidant properties. We evaluated the serum levels of inflammation markers serum amyloid A (SAA) and monocyte chemoattractant protein-1 (MCP-1) in 44 HS patients and 16 healthy controls. Additionally, we assessed the activity of the antioxidant enzyme paraoxonase-1 (PON1) associated with HDL, as well as the HDL redox properties using a cell-free method. HS patients showed significantly higher serum levels of MCP-1 and SAA compared to controls. PON1 activity was considerably lower in HS patients, suggesting impaired antioxidant functions of HDL. These changes in HDL correlated with the severity of HS but occurred without significant alterations in plasma HDL levels. Our findings indicate that inflammation and oxidative stress could contribute to the dysfunction of HDL in HS patients. Identifying dysfunctional HDL could provide valuable insights into the pathogenesis of HS and its associated complications, offering potential targets for new therapeutic strategies.

## 1. Introduction

Hidradenitis suppurativa (HS) is a chronic, recurrent, inflammatory, immune-mediated skin disease that originates in the pilosebaceous unit, with subsequent involvement of the associated apocrine glands [1]. The prevalence of HS ranges from 1% to 4%, with a female to male ratio of 3:1 and a usually post-pubertal onset [2]. HS lesions, consisting of inflammatory nodules, abscesses, and sinus tracts, affect mostly the skin folds such as the axillary, inguinal, perianal, and inframammary areas [3]. The pain, unpleasant odor, and unaesthetic appearance associated with the lesions can lead to discomfort and social isolation, with an adverse impact on patients’ quality of life [4]. HS management is challenging and includes medical therapy, surgical approach, and lifestyle modifications. The medical treatment primarily includes topical therapy, systemic antibiotics, retinoids, oral zinc, and antiandrogen therapy [5,6,7,8,9,10]. Although several novel biologics and small molecules are currently being investigated, adalimumab (anti-TNF-α) and the interleukin-17 inhibitors bimekizumab and secukinumab are the only approved biologic agents for the treatment of HS [11,12]. HS is a complex, underestimated, and not completely understood disease in which the genetic predisposition, particularly the dysregulation of the gamma-secretase/Notch pathway, combines with exogenous triggering factors such as smoking and obesity [13,14,15].

Oxidative stress is the result of an imbalance between the production of oxidant species, particularly reactive oxygen species (ROS), generated during physiological aerobic metabolism and pathological inflammatory processes, and their elimination through antioxidant defense mechanisms. The excessive production of ROS in the cells and tissues and/or the reduction of their degradation through the antioxidant system can lead to the damage of cellular molecules, including DNA, proteins, and membrane lipids [16]. Recent studies suggest that oxidative stress may be implicated in the pathogenesis of several inflammatory immune-mediated skin disorders such as psoriasis, atopic dermatitis, and acne [17,18,19,20,21,22].

High-density lipoproteins (HDL), which regulate cellular metabolism, cholesterol uptake and immune response, are strictly involved in inflammatory processes and oxidative stress associated with immune-mediated cutaneous diseases [23,24,25,26,27].

Paraoxonase 1 (PON1) has a crucial role in the anti-inflammatory and antioxidant properties of HDL. PON enzymes (PON1, PON2, and PON3) are calcium-dependent enzymes with antioxidant properties. PON1, which is one of the most significant antioxidant defense systems against lipid peroxidation, is synthetized in the liver and released into the circulation in association with HDL. Specifically, PON1 protects membranes, HDL, and the low-density lipoprotein (LDL) from lipid peroxidation caused by ROS [28,29,30,31,32].

A crucial process in the development of inflammatory diseases is exerted by monocytes that migrate to the site of inflammation and differentiate into macrophages when the chemokine (C-C motif) receptor (CCR2) interacts with the chemokine (C-C motif) ligand 2 (CCL2), also referred to as monocyte chemoattractant protein 1 (MCP-1) [33].

HDL-associated PON1 may exert anti-inflammatory effects by inhibiting the production of MCP-1 and reducing monocyte recruitment to the arterial wall [34,35,36]. Under certain circumstances, HDL lose their atheroprotective properties, resulting in the formation of dysfunctional HDL particles. Dysfunctional HDL particles are characterized by modifications in lipid composition and protein content and have been observed in patients affected by chronic diseases associated with oxidative stress [37,38,39].

Serum amyloid A (SAA) is among the plasma proteins that modulate the antioxidative properties of HDL, contributing to their dysfunction. SAA is a highly sensitive acute-phase reactant that has been linked to several chronic inflammatory diseases [40].

Alteration of plasma lipid and lipoproteins induced by oxidative stress and modification of PON1 activity have been deeply investigated in several inflammatory skin diseases, including psoriasis and atopic dermatitis, and in various HS comorbidities such as metabolic disorders and cardiovascular disease [18,21,41]. Conversely, there are currently no data on the role of lipid peroxidation, PON1 activity, and oxidative stress in HS.

The aim of the present study was to evaluate the association between chronic inflammation, impaired HDL function, and elevated SAA and MCP-1 levels in HS. Therefore, PON1 activity and markers of inflammation, such as SAA and MCP-1, were examined in the serum of HS patients compared with healthy controls. The antioxidant properties of HDL were also analyzed by a cell-free method. Moreover, the relationship between lipid profile, biochemical markers, and disease severity was investigated.

## 2. Methods

### 2.1. Patients

44 patients affected by HS (32 females and 12 males with a mean current age of 32.5 ± 2.0) were included through consecutive recruitment from new referrals and follow-up visits at the Clinic of Dermatology of the Department of Clinical and Molecular Sciences of the Polytechnic University of Marche. Inclusion criteria for patients were male and female patients ≥ 18 years of age at the time of enrollment, diagnosis of HS (mild, moderate, or severe) based on clinical history and physical and ultrasonographic examination for at least 6 months, patients without other immune-mediated inflammatory conditions (e.g., psoriasis, inflammatory bowel disease, rheumatologic disorders).

The clinical severity of HS was assessed using the International Hidradenitis Suppurativa Severity Score System (IHS4), considering IHS4 ≤ 3 mild HS, from 4 to 10 moderate HS, and ≥11 severe HS [42]. In total, 16 subjects, age and sex matched (11 females and 5 males with a mean current age of 34.1 ± 3.8) without skin or systemic inflammatory disease and without a family history of HS were also recruited. All the procedures were in accordance with the Helsinki Declaration of 1975, as revised in 2000. The study was approved by the “Ethics Committee, Azienda Ospedaliero Universitaria (AOU) delle Marche”. Written informed consent was obtained from all participants before their enrollment in the study.

### 2.2. Sample Collection

Morning blood samples were collected after an overnight fast from both controls and HS patients using a Venflon catheter, processed according to the manufacturer’s instructions (BD Vacutainer^®^ tubes, Becton, Dickinson and Company (DB), Franklin Lakes, NJ, USA), and centrifuged at 3000× *g* and +4 °C for 10 min. The serum obtained was stored at −20 °C until analysis. Fasting blood glucose and blood lipids were evaluated at the accredited laboratory of the Marche AOU.

### 2.3. Levels of Serum Amyloid Protein (SAA)

SAA levels in serum were quantified using a human serum amyloid A ELISA kit (RAB0420, Millipore—Saint Louis, MO, USA). Optical densities were measured at 450 nm using a 96-well microplate reader (Synergy HT; BioTek, Winooski, VT, USA). The concentrations were calculated from the standard curve generated by a four-parameter curve-fitting program. Sensitivity of SAA measurements was ≤500 pg/mL, and intra- and inter-assay coefficients of variation were <10% and <12%, respectively.

### 2.4. Levels of Serum Monocyte Chemoattractant Protein 1 (MCP-1)

MCP-1 protein levels in serum from study participants were measured using a solid-phase sandwich enzyme-linked immunosorbent assay (Human MCP-1/CCL2 ELISA Kit, RAB0054, Millipore—Saint Louis, MO, USA). Optical densities were measured at 450 nm using a 96-well microplate reader (Synergy HT; BioTek, Winooski, VT, USA). The concentrations were calculated from the standard curve generated by a four-parameter curve-fitting program. Intra- and inter-assay coefficients of variation were <4.9% and <12.9%, respectively.

### 2.5. Paraoxonase 1 (PON1) Activities

PON1 is considered a “promiscuous” enzyme since it can hydrolyze different substrates such as organophosphate compounds, non-phosphorus arylester, and lactones, which are considered its physiological substrates. Therefore, paraoxonase, arylesterase, and lactonase activities were evaluated in serum, using as substrates paraoxone, phenylacetate, and dihydrocoumarin, respectively.

All enzymatic assays were conducted using 96-well microplates, with each reaction carried out in a final volume of 200 µL. Control wells were included on each plate to account for spontaneous substrate hydrolysis [43].

Paraoxonase (PON) Activity. The basal assay mixture included 50 mM glycine/NaOH with a pH of 10.5, 1 mM CaCl_2_, and 2.0 mmol/L paraoxon. For each reaction, 5 µL of undiluted serum was used in a final volume of 200 µL. Paraoxon hydrolysis was spectrophotometrically monitored for 8 min (every 15 s) at 412 nm. One unit of paraoxonase activity was equivalent to one nmol of paraoxon hydrolyzed/min/mL.

Arylesterase (ARE) Activity. Serum samples were diluted 1:10 with 50 mM Tris-HCl with a pH of 8.0 and 1 mM CaCl_2_, and then 5 µL was taken for a total reaction volume of 200 µL. After the addition of the substrate phenyl acetate (1 mmol/L), the hydrolysis was monitored at 270 nm for 3 min (every 15 s). One unit of arylesterase activity was equivalent to one µmol of phenyl acetate hydrolyzed/min/mL.

Lactonase (LAC) Activity. Serum was diluted at a ratio of 1:10 in 50 mM Tris-HCl buffer (pH 7.5) with 1 mM CaCl_2_. An aliquot of 3 µL was used for the assay. After the addition of the substrate dihydrocoumarin (DHC) (1.0 mM), the hydrolysis was monitored at 270 nm for 10 min (every 15 s). One unit of lactonase activity was equivalent to one µmol of DHC hydrolyzed/min/mL.

### 2.6. Evaluation of the Functional Properties of HDL

HDL redox activity was assessed using a fluorometric biochemical cell-free assay [43,44].

This assay evaluates the effect of HDL on the oxidation rate of the fluorogenic substrate dihydrorhodamine 123 (DHR). HDL particles were extracted from serum through selective precipitation using polyethylene glycol. For the quantitative determination of the cholesterol content of HDL, the Chema kit (Chema Diagnostica, Monsano, AN, Italy.) was used. A stock solution of DHR (50 mM) was diluted at 1:1000 in iron-free N-2-hydroxyethylpiperazine-N-2-ethanesulfonic acid-buffered saline (HBS; HEPES 20 mM, NaCl 150 mM, pH 7.4) prepared as previously described. In a 96-well plate, aliquots of HDL (1.25 μg HDL-cholesterol) and DHR working solution (final concentration of 7 μM) were added, and the volume was diluted to 200 μL with HBS buffer. Right after the addition of DHR, the plate was protected from light and inserted into a fluorescence microplate reader. Fluorescence measurements were taken every 2 min over the course of 1 h using a Synergy HT microplate reader (BioTek, Winooski, VT, USA) using a 485/538 nm excitation/emission filter. The value was calculated as the mean of quadruplicates for the wells containing only DHR and for samples containing DHR and individual samples. The DHR oxidation rate (DOR) was calculated for each well as the slope for the linear regression of fluorescence intensity between 10 and 60 min and was expressed as fluorescence units per minute (FU/min). The value was calculated as the mean of quadruplicates for the wells containing only DHR and for samples containing DHR and individual samples.

### 2.7. Statistical Analysis

Statistical analysis was performed with GraphPad Prism 8.2 software (GraphPad, San Diego, CA, USA). All data are expressed as means ± S.E.M. The normality of the data was assessed using the Shapiro–Wilk test. For the comparison of normally distributed variables between groups, Student’s *t*-test was used. Mann–Whitney U test was used to compare variables not normally distributed. Quantitative values were compared using either the Student’s *t*-test or the Mann–Whitney U test. Correlations between clinical and biochemical parameters were assessed using Spearman’s correlation test. A *p* value ≤ 0.05 was considered statistically significant. A correlation matrix was obtained using the Displayr platform for survey data analysis (https://www.displayr.com, accessed on 20 September 2023).

## 3. Results

### 3.1. Subjects

Clinical characteristics and plasma lipids of controls and subjects affected by HS are summarized in Table 1. 9% of patients were classified as having mild disease (*n* = 4, IHS4 ≤ 3), 34% of patients as having moderate disease (*n* = 15, 4 < IHS4 < 10) and 57% of patients as having severe HS (*n* = 25, IHS4 ≥ 11). IHS4 was not found to be correlated with disease duration. In all, 38.1% were normal-weight patients, 33.3% were overweight patients, and 28.6% were obese patients. Comorbidities included acne (34.1%) and pilonidal disease (38.6%). No significant correlation was observed between the BMI of patients and IHS4 values. A similar distribution of BMI was observed in controls. Plasma levels of triglycerides (TG), total cholesterol (TC), HDL-C, and LDL-C were not significantly different in HS patients compared to controls.

### 3.2. Inflammation Markers and HDL Functions

**Figure 1:**
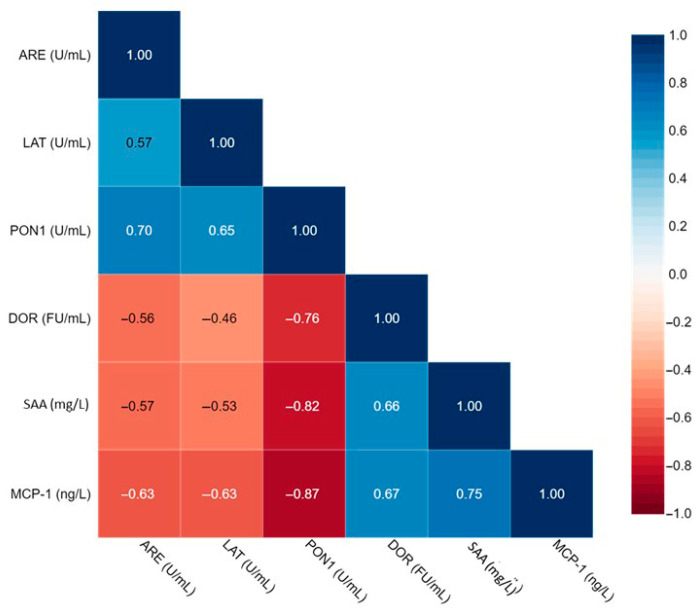
Correlation matrix for biochemical parameters. Values inside each box represent Spearman’s correlation coefficient. ARE, PON1 arylestrase activity; LAT, PON1 lactonase activity; PON1, paraoxonase 1 activity; DOR, dihydrorhodamine oxidation rate; SAA, serum amyloid A; MCP-1, monocyte chemoattractant protein 1.

As reported in Table 2, significantly higher levels of SAA and MCP-1 were observed in the serum of the whole group of HS patients compared to controls. The study of HDL functionality evaluated using the activities of PON1 enzyme (paraoxonase, arylesterase, and lactonase) and HDL redox activity revealed significant differences. Lower activities of PON1 were observed in the serum of HS patients compared to control subjects (*p* < 0.05) (Table 2). HDL redox activity, assessed as DOR (oxidation rate of DHR), was significantly higher in HDL isolated from the serum of HS subjects compared to controls (*p* < 0.001) (Table 2). All these results suggest a pro-inflammatory status and alteration of HDL properties in HS patients. This hypothesis is supported by the pairwise correlation between the biochemical parameters calculated by including both HS patients and controls using Spearman correlation coefficients. A significant negative correlation was established between HDL redox activity and the arylesterase (r = −0.56, *n* = 60, *p* < 0.0001), lactonase (r = −0.46, *n* = 60, *p* < 0.0001), and paraoxonase (r = −0.76, *n* = 60, *p* < 0.0001) activities of PON1. HDL redox activity was also positively correlated with serum levels of SAA (r = 0.66, *n* = 60, *p* < 0.0001) and MCP-1 (r = 0.67, *n* = 60, *p* < 0.0001). Furthermore, negative significant correlations were established between the catalytic activities of PON1 and serum levels of SAA and MCP-1 (Figure 1). All these results indicate that HDL function is closely related to PON1 and that SAA and MCP-1, widely used as biochemical markers of inflammation, are also related to HDL functions.

### 3.3. Relationship Between Biochemical Parameters and Clinical Parameters

To examine the correlation between alterations in HDL function and levels of inflammatory markers and disease severity, HS patients were divided into two groups based on the IHS4. The mild/moderate group included patients with IHS4 < 10 (*n* = 19); the severe group included HS patients with IHS4 ≥ 11 (*n* = 25).

**Figure 2:**
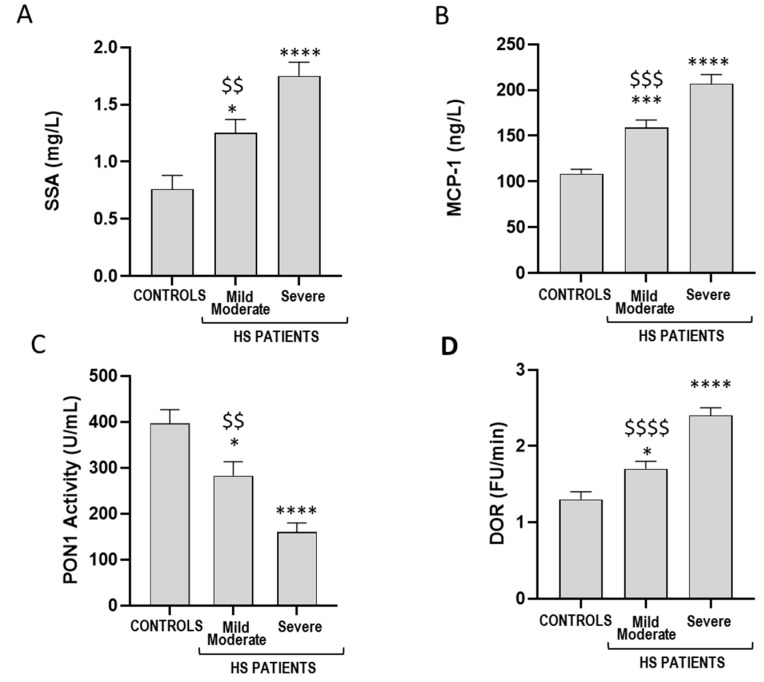
HDL function and levels of inflammatory markers in serum of controls and patients with mild/moderate HS (*n* = 19, IHS4 ≤ 10) and severe HS (*n* = 25, IHS4 ≥ 11). Serum levels of serum amyloid A (SAA) (**A**); serum levels of monocyte chemoattractant protein (MCP-1) (**B**); paraoxonase 1 (PON1) activity (**C**); HDL redox activity expressed as DOR, dihydrorhodamine oxidation rate (**D**). * *p* < 0.05 vs. controls, *** *p* < 0.001 vs. controls, **** *p* < 0.0001 vs. controls, $$ *p* < 0.01 vs. severe HS, $$$ *p* < 0.001 vs. severe HS, $$$$ *p* < 0.0001 vs. severe HS.

As shown in Figure 2, patients with severe HS showed higher serum levels of SAA and MCP-1 compared to the mild/moderate group. Moreover, a lower PON1 paraoxonase activity (Figure 2C) and HDL characterized by a higher redox activity (Figure 2D) were observed in HS patients with IHS4 ≥ 11.

## 4. Discussion

HDL particles, widely recognized for their utility in reverse cholesterol transport and physio-pathological roles in cardiovascular diseases, also exert important antioxidant and anti-inflammatory properties as they inhibit LDL oxidation, cytokine-induced production of endothelial cell adhesion molecules, and ox-LDL-induced MCP-1 production by endothelial cells, thereby modulating inflammatory processes through different molecular mechanisms [23,24,34,35]. A growing interest is dedicated to the potential roles of HDL in the molecular mechanisms of inflammatory skin diseases. There is strong evidence that HDL considerably affects the function of various immune cells, and alterations of HDL antioxidant activities have been shown in patients with atopic dermatitis, psoriasis, and other skin diseases as reviewed [23,24,25,26,27].

HS is a chronic, inflammatory, immune-mediated skin disorder with a complex and multifactorial pathogenesis, associated with several comorbidities belonging to the cardio-metabolic sphere such as overweight/obesity, hyperlipidemia, and cardiovascular complications [45,46,47,48,49].

Previous studies have demonstrated that HS shares mutual pathophysiology with other inflammatory skin diseases [50]. In fact, alterations of keratinocytes and upregulation of several cytokine expressions have been demonstrated [51,52]. In addition, plasma lipid alterations have been described in HS patients, with an association between hypertriglyceridemia and low HDL cholesterol [46,53]. Conversely, the relationship between inflammation and HDL alterations as well, as oxidative stress, has not yet been investigated in HS.

PON1, an HDL-associated enzyme, is extensively recognized for its protective effects against oxidative modifications of cell membranes and lipoproteins, and for its anti-inflammatory and anti-atherogenic functions [28,29,30,31,32]. Given the importance of modulating the inflammatory response in HS, PON1 activities (paraoxonase, arylesterase, and lactonase) were analyzed in the serum of HS patients. We observed a significant decrease in PON1 activity in the serum of HS patients compared to controls, denoting impaired HDL functionality in patients with HS. In addition, lower PON1 paraoxonase activity was observed in patients with severe HS compared to the mild-moderate group, suggesting a correlation between reduced PON1 activity and disease severity. Alterations in HDL were also confirmed by data from the cell-free method used to assess the redox properties of HDL, which revealed a greater tendency for HDL from HS patients to be oxidized, especially in patients with severe disease.

Chemokines, including MCP-1, also play a central role in the chronic inflammation observed in HS, being involved in the recruitment of monocytes to the affected skin regions, increasing the subsequent infiltration of immune cells and the release of pro-inflammatory cytokines [54]. In our study, we found a significant increase in serum levels of MCP-1 in patients with HS compared to controls, confirming what has been reported on other inflammatory skin diseases such as psoriasis and atopic dermatitis, and in sweat samples from HS patients [55,56,57]. Moreover, an increase in SAA was also detected in HS patients compared to controls.

It is well known that both MCP-1 and SAA are closely related with HDL and PON1. MCP-1 is upregulated after pro-inflammatory stimuli and tissue damage, and it has been reported to trigger endoplasmic reticulum stress and autophagy [58]. Karabacak et al. reported a negative correlation between plasma MCP-1 levels and HDL-C levels [59]. A relationship between changes in PON1 status and MCP-1 concentrations has also been shown in patients with severe sepsis [60].

A recent study by Navrazhina et al. revealed that several pro-inflammatory molecules are overexpressed in the skin of HS subjects, including MCP-1. It has been suggested that the higher protein products in the skin can result in the release and potential diffusion into the blood [61]. Further studies are necessary to investigate whether HDL can modulate MCP-1 secretion in keratinocyte of HS patients and the underlying molecular mechanisms.

The increase in SAA in HS subjects, observed in the present study, is in accordance with previous studies [62,63]. SAA, an acute-phase protein, is a reliable clinical marker of inflammation. Elevated SAA concentrations are believed to contribute to the pathophysiological processes underlying atherosclerosis, particularly by promoting endothelial dysfunction [64]. In addition, SAA may drive the conversion of HDL into a dysfunctional form. SAA can bind to the surface of HDL, displacing ApoA1 and decreasing the binding affinity for PON1 [38,39,65,66,67]. These alterations impair HDL functionality and its protective effects.

The negative correlation between PON1 activity, HDL redox activity, and the serum levels of MCP-1 and SAA in both HS patients and controls indicates that elevated levels of SAA and MCP-1 are linked to impaired HDL function. These findings collectively underscore the complex interplay between chronic inflammation and altered antioxidant and anti-inflammatory function of HDL, with potential implications for cardiovascular risk in inflammatory skin diseases like HS. The correlation between the severity of HS and inflammation markers (MCP-1 and SAA) as well as HDL functionality (PON1 activity and HDL redox activity) highlights the potential contribution of dysfunctional HDL to the progression of HS.

**Figure 3:**
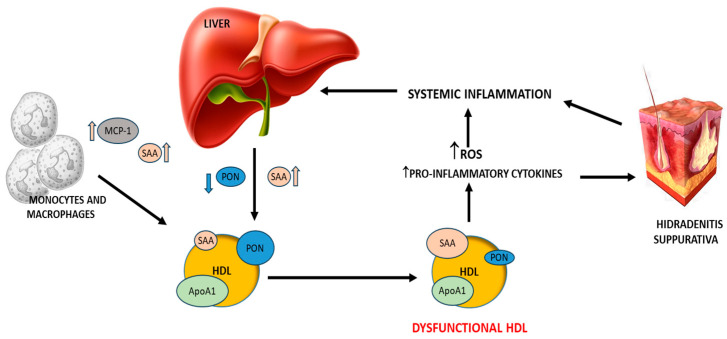
Systemic inflammation induces SAA secretion and reduces PON1 synthesis by the liver via cytokine signaling. SAA and inflammatory cytokines, including MCP-1, can also be produced by monocytes and macrophages. SAA associates with HDL surface displacing PON1 and ApoA1. HDL enriched in SAA and depleted of ApoA1 and PON1 shows a lower antioxidant and anti-inflammatory capacity. Dysfunctional HDLs may contribute to pro-oxidant and pro-inflammatory processes with consequent exacerbation of the chronic inflammation condition in HS. SAA, serum amyloid A; PON1, paraoxonase-1; MCP-1, monocyte chemoattractant protein.

Figure 3 summarizes the complex interaction between HDL and inflammation, underlining their potential roles in HS. According to existing literature, systemic inflammation promotes hepatic secretion of serum SAA and downregulates PON1 synthesis through cytokine signaling. In addition, both SAA and inflammatory cytokines, including MCP-1, can be produced by monocytes and macrophages. Previous studies have shown that high plasma levels of SAA lead to their incorporation into HDL particles, resulting in the displacement of key functional components such as PON1 and apolipoprotein A1 (ApoA1). As demonstrated by in vitro and ex vivo studies, HDL particles enriched in SAA and depleted of ApoA1 and PON1 exhibit lower antioxidant and anti-inflammatory properties [38,39,65,66,67]. Dysfunctional HDL may therefore contribute to pro-oxidant and pro-inflammatory processes with consequent exacerbation of the chronic inflammation in HS.

The main limitation of our study is the restricted number of patients and controls, particularly after stratification by disease severity, which may have affected the statistical power of subgroup analyses. Our preliminary insights should be further validated in larger, well-powered cohorts.

## 5. Conclusions

Most inflammatory skin diseases share common biochemical mechanisms. Oxidative stress, inflammation, and HDL alterations play a central role in the pathophysiology of these diseases. Our study emphasizes the role of inflammation in driving HDL dysfunction in HS patients. Elevated levels of the inflammatory markers SAA and MCP-1 are associated with impaired HDL functionality, including reduced PON1 activity and increased HDL redox activity. The alterations of HDL have been observed in the absence of significant changes in HDL-cholesterol levels. The alterations in HDL may contribute to the exacerbation of oxidative stress and chronic inflammation, potentially playing a significant role in the progression of HS. The relationship between disease severity and SAA and MCP-1 levels emphasizes the importance of HDL dysfunction in HS and suggests that targeting HDL-related pathways could offer potential therapeutic strategies for managing the condition.

## Figures and Tables

**Table 1:** Clinical characteristics and plasma parameters in controls and subjects affected by HS.

	Controls (*n* = 16)	HS Patients (*n* = 44)
Age (years)	34.1 ± 3.8	32.9 ± 2.0
BMI (kg/m^2^)	26.3 ± 0.8	27.1 ± 0.8
IHS4	-	14.4 ± 1.2
Age of onset (years)	-	16.8 ± 0.8
TG (mg/dL)	107.1 ± 11.2	104.7 ± 9.2
TC (mg/dL)	184.7 ± 6.4	171.4 ± 4.9
LDL-C (mg/dL)	105.9 ± 6.1	102.3 ± 4.1
HDL-C (mg/dL)	52.7 ± 3.4	49.5 ± 1.6

BMI, Body mass index; IHS4, International Hidradenitis Suppurativa Severity Score System; TG, triglycerides; TC, total cholesterol; LDL-C, LDL cholesterol; HDL-C, HDL cholesterol. Values are expressed as mean ± S.E.M.

**Table 2:** Inflammation markers, paraoxonase 1 (PON1) activities, and HDL redox activity in serum of controls and total HS patients.

	Controls (*n* = 16)	HS Patients (*n* = 44)
SAA (mg/L)	0.76 ± 0.12	1.52 ± 0.10 ***
MCP-1 (ng/L)	108.6 ± 4.8	186.6 ± 7.5 ***
PON1-paraoxonase (U/mL)	396.3 ± 30.3	213.8 ± 19.0 ***
PON1-arylesterase (U/mL)	107.5 ± 4.2	79.1 ± 2.9 ***
PON1-lactonase (U/mL)	73.7 ± 8.8	40.1 ± 3.1 ***
DOR (FU/min)	1.3 ± 0.1	2.1 ± 0.1 ***

SAA, serum amyloid A; MCP-1, monocyte chemoattractant protein 1; PON1, paraoxonase-1; HDL redox activity is expressed as DOR, dihydrorhodamine oxidation rate. Values are expressed as mean ± S.E.M. *** *p* < 0.0001 vs. controls.

## Data Availability

Data is contained within the article.
